# A Long-Term Lens: Cumulative Impacts of Free-Roaming Cat Management Strategy and Intensity on Preventable Cat Mortalities

**DOI:** 10.3389/fvets.2019.00238

**Published:** 2019-07-26

**Authors:** John D. Boone, Philip S. Miller, Joyce R. Briggs, Valerie A. W. Benka, Dennis F. Lawler, Margaret Slater, Julie K. Levy, Stephen Zawistowski

**Affiliations:** ^1^Great Basin Bird Observatory, Reno, NV, United States; ^2^Conservation Planning Specialist Group, Species Survival Commission, International Union for Conservation of Nature, Apple Valley, MN, United States; ^3^Alliance for Contraception in Cats and Dogs, Portland, OR, United States; ^4^Illinois State Museum, Springfield, IL, United States; ^5^Strategy and Research Department, American Society for the Prevention of Cruelty to Animals, Florence, MA, United States; ^6^Maddie's Shelter Medicine Program, Department of Small Animal Clinical Sciences, University of Florida, Gainesville, FL, United States; ^7^Animal Behavior and Conservation Program, Hunter College, New York, NY, United States

**Keywords:** free-roaming cats, trap-neuter-return, cat management, population dynamics, simulation model, lifesaving

## Abstract

This study used a previously developed stochastic simulation model (1) to estimate the impact of different management actions on free-roaming kitten and cat mortality over a 10-year period. These longer-term cumulative impacts have not been systematically examined to date. We examined seven management scenarios, including: (1) taking no action, (2) low-intensity removal, (3) high-intensity removal, (4) low-intensity episodic culling, (5) high-intensity episodic culling, (6) low-intensity trap-neuter-return (TNR), and (7) high-intensity TNR. For each scenario we tracked within the model the number of kittens born, the number of kittens surviving to adulthood, and the number of adults removed using lethal control over the entire 10-year simulation. We further defined all kitten deaths and lethal removal of adults as “preventable” deaths because they could potentially be reduced by certain management actions. Our simulation results suggested that the cumulative number of preventable deaths over 10 years for an initial population of 50 cats is highest for a “no-action” scenario, estimated at 1,000 deaths. It is lowest for a high-intensity TNR scenario, estimated at 32 deaths, a 31-fold difference. For all management scenarios tested, including removal and culling, the model predicted fewer preventable deaths than for a no-action scenario. For all management scenarios, the model predicted that the higher-intensity option (defined in terms of the proportion of animals sterilized or removed within a given time period) would result in fewer preventable deaths over time than the lower-intensity option. Based on these findings, we conclude that management intensity is important not only to reduce populations more quickly, but also to minimize the number of preventable deaths that occur over time. Accordingly, the lessons for the animal welfare community are both encouraging and cautionary. With sufficient intensity, management by TNR offers significant advantages in terms of combined lifesaving and population size reduction. At lower intensity levels, these advantages are greatly reduced or eliminated. We recommend that those who seek to minimize suffering and maximize lifesaving for free-roaming cats attempt to balance prospective goals (i.e., saving lives tomorrow) with proximate goals (i.e., saving lives today), and recognize that thoughtful choice of management strategies can ensure that both of these complementary goals are achieved.

## Introduction

Trap-neuter-return (TNR) programs vary substantially in scope, scale, intensity, and duration, but most employ a combination of sterilizing, vaccinating, feeding, and caring for free-roaming cats. Specific goals of TNR programs can include population stabilization or reduction (1–6); reducing shelter admissions, crowding, and deaths (7); mitigating nuisance behaviors (8, 9); reducing predation on wildlife (10); improving cat welfare (11, 12); and reducing numbers of cats that die from the risks and hardships of living outdoors (5, 11–15).

The results of TNR programs are most commonly quantified by the number of cats sterilized. Other metrics that may be considered include the numbers of cats returned to the point of origin, vaccinated, or fed, as well as indicators of health [see (15–17) for examples]. Less commonly, changes in population size may be tracked as an indicator of impact (18). What is rarely considered is that changes in the numbers of births, deaths, and immigration events that may result from management efforts could have multiplicative consequences that—over time—outweigh the more obvious and immediate management impacts.

Longer-term cumulative effects (defined in this model as effects occurring over a 10-year period) of different free-roaming cat management approaches have not been explored systematically, and little guidance exists to address these prospective concerns when creating and evaluating management strategies and goals. In this paper, we estimate the cumulative demographic consequences and the population end points of several different cat population management approaches that are currently available, including TNR, using a published simulation model of free-roaming cat population dynamics (1). We relate these outcomes to “lifesaving,” a focal concept in the animal welfare field^1^^,^^2^ (19), and specifically to the number of “preventable” deaths that occur under different management scenarios. We define preventable deaths as those that could likely be reduced or eliminated using an alternative population management approach, specifically the deaths of kittens under 6 months old that fail to reach adulthood, and the deaths of any cats due to lethal management.

Although there is considerable diversity and complexity to stakeholder views, public debate about free-roaming cat management and policy has been polarized and sometimes antagonistic (10, 13, 14, 20–23). One set of stakeholders prioritizes quickly and permanently eliminating outdoor cat populations, by lethal means if necessary (13, 14, 20, 22, 23). This position is often motivated by concerns about cat predation on native wildlife species and threats of disease transmission. Another cohort of stakeholders prioritizes non-lethal management, including TNR. These proponents often emphasize that TNR has the capacity to successfully reduce and stabilize cat populations in a humane fashion over time, in addition to meeting animal welfare goals (11, 12).

In this analysis, we use a predictive simulation model to evaluate the relative effectiveness of different population management strategies for free-roaming cats in terms of both cumulative preventable deaths and population size reduction. We then consider the implications of these results for establishing best management practices. Specifically, we explore whether current competing paradigms of cat management could become more convergent and possibly synergistic when viewed from a longer-term perspective. If so, then combining these goals into a more integrated paradigm for “best management practices” at realistic time scales could lead to better outcomes for cats at the individual and population levels, mitigate predation risk to wildlife, and reduce conflict among stakeholders.

## Methods

In 2014, we developed an individual-based stochastic model to simulate free-roaming cat population dynamics using the software package *Vortex* version 9.99b (24) to estimate the demographic outcomes associated with various management scenarios (1). We used this model, now updated to *Vortex* version 10.2 (25), to generate the new results that are presented in this report. Model details are detailed in Miller et al. (1) and summarized briefly here.

The model is structured as a series of sequential 6-month time steps. During each time step, probabilistic age-specific birth and death rates are applied to each individual in the simulated population, along with specified management actions. These operations result in changes to population size and age-sex structure that collectively define the starting point for the next time step. Model parameters such as birth and death rates were determined by literature review or expert judgment (1) to reflect typical population function, and management scenarios were defined *a priori* to reflect a realistic range of possibilities. In addition, kitten mortality was structured to increase as the population approached its carrying capacity, as higher population density will create more stressful conditions (e.g., greater disease transmission, more competition for food) that will result in more individuals dying within 6 months after being born (see also Supplementary Materials).

Unlike most simulation models for free-roaming cats (26–30), our model incorporated demographic connectivity between our “focal” population and cats in surrounding areas by allowing dispersal (consisting of both immigration into and emigration out of a given population) and abandonment of owned pet cats to occur probabilistically. Immigration rates averaged 2% of the extant source population (comprised of individuals aged 0.5–2 years and 75% male) per time step, and abandonment rates averaged one litter with approximately three surviving kittens per time step. Population dynamics that were not explicitly incorporated into the model included more complex forms of density dependence, differential longevity of sterilized cats (31), and postulated differential male fecundity mediated by social stratification (32, 33). The 6-month time step was not intended to suggest that births, deaths, or management actions do or should occur at these time intervals, but represented a temporal resolution that in our judgment best matched available field data [e.g., seasonal breeding documented in (17, 34)] and balanced computational tractability with biological realism. A summary of baseline model input parameter values is presented in Table 1.

**Table 1:** Summary of numerical input values for baseline demographic models.

**Model input parameter**	**Baseline value**
**GENERAL MODEL SETUP**	
Model timestep	6 months
Number of iterations for each scenario	1,000
Number of populations	2: Focal population, Neighborhood
**POPULATION DEMOGRAPHICS**	
Initial population size	Focal population = 50; Neighborhood = 200
Sex ratio (initial population and new litters)	50:50
Age of first breeding	6 months (females and males)
Female breeding rate (producing litters)	48% (“winter”); 92% (“summer”)
Average annual number of litters per female	1.4
Average litter size	3.5
Male breeding rate	100% of intact males available for breeding
Kitten mortality to 6 months	75% (low density) to 90% (high density)
Adult mortality (6-month interval)	5.2% (10% annually)
**METAPOPULATION STRUCTURE**	
Disperser characteristics	Age 6–24 months; 75% male
Mean dispersal rate per timestep	2% of source population size
Cost to dispersal (survival rate)	75% survival of dispersers
Litter abandonment (per timestep)	Mean of 3 kittens into focal population

For this analysis, we simulated a set of discrete management scenarios over a 10-year (or 20-time-step) period. Although these scenarios do not represent all management options, they do represent typical approaches for which real-world precedents exist, particularly in the municipal settings where a blend of removal, culling, and TNR programs may co-exist. Simulations began with the focal and neighborhood populations composed of 50 and 200 cats, respectively. Individuals in these populations were initially distributed across age- and sex-class according to the stable age distribution, which is calculated automatically by the model in accordance with the stated reproductive and survival rates. With this initialization procedure, no long-term model “burn-in” was necessary and simulation results were not adversely biased by non-steady-state demographic dynamics. Furthermore, we assume that each population is at its maximum long-term abundance within its given habitat; in other words, each of the populations are at the ecological carrying capacity, where growth beyond this abundance cannot be sustained by the available local resources (see Supplementary Materials).

The focal population was tracked over time as it changed due to management and other factors. The focal population was surrounded by a larger “neighborhood” population of 200 cats that was not managed and provided a source of potential immigrants.

The following scenarios were simulated:

1. No action: In this scenario, no attempt was made to manage the focal population. It provided a baseline against which other active management scenarios were compared.
2. Remove-low intensity: This scenario involved trapping and removing 25% of the cats in the focal population during each time step. We assumed for this analysis that these cats were euthanized after removal, though we recognize that adoption could be an alternative in some real-world settings. Because the number of cats in the population changed over time, the number of cats removed during each time step varied over time. This scenario approximates ongoing, steady removal of free-roaming cats by an animal control agency.
3. Remove-high intensity: This scenario is identical to “remove-low intensity,” except that 50% of the cats in the focal population were removed (and assumed to be euthanized) during each time step. This scenario approximates the eradication programs that are sometimes pursued in protected wildlife habitat.
4. Cull-low intensity: This scenario involved removing and euthanizing 25% of the cats present in the population during the initial time step, and then taking no action until the population recovered to its carrying capacity over several time steps, at which point another 25% cull was performed. This cycle was repeated throughout the 10-year period. This represents the episodic removals that may be conducted by animal control agencies in response to nuisance complaints or other concerns.
5. Cull-high intensity: This scenario is identical to “cull-low intensity,” except that episodic culls removed 50% of the existing population.
6. Sterilize-low intensity: This is a TNR scenario in which 25% of the intact (i.e., non-sterilized) cats in the focal population were trapped, sterilized, and returned during each time step. Because the number of intact cats in the population changed over time, the number of cats trapped and sterilized varied across time steps. This scenario reflects the lower-intensity TNR efforts that sometimes occur. Given the influx rates we structured in the model, this level of sterilization intensity is expected to eventually generate a sterilization rate of ~60% over most of the simulation period, which leads to a small population size reduction over time.
7. Sterilize-high intensity: This scenario is the same as “sterilize-low intensity,” except that 75% of the intact cats present in the population were trapped, sterilized, and returned during each time step. This scenario represents the higher-intensity “targeted” TNR programs that occur in some areas (15, 35, 36). Given the influx rates we structured in the model, this management intensity generates a sterilization rate of over 80% throughout nearly all of the simulation trajectory, which reduces initial population size by about half over time.

The word “intensity” is hereafter omitted from scenario names for brevity.

For each of these scenarios, 1,000 model iterations were performed, with each iteration generating a unique set of results due to the stochastic variability in demographic factors that define the model structure (1). For each iteration, we tracked multiple output metrics on a time-step basis, which included:

1. Number cats removed or sterilized;
2. Number of kittens born locally;
3. Net number of cats that disperse or are abandoned into the focal population (total cats coming in minus those that emigrate out);
4. Number of cats present at the beginning and end of the time step, categorized by age and sterilization status;
5. Number of kittens (cats under 6 months of age) and adults that die of “natural” causes, which excludes cats that are euthanized as part of a management scenario.

Final population size after 10 years was determined by computing the average number of living cats across all 1,000 iterations in each scenario at the end of the last time step. For computational tractability, each of the output metrics listed above was averaged across a random subset of 100 iterations for each time step and each scenario, and then summed over all time steps to produce cumulative outcome estimates for each management scenario. As a basis for comparing cumulative management outcomes, we identified two specific types of mortality that could be tracked in the model and that we assumed were undesirable from an animal welfare perspective: (1) deaths of kittens prior to reaching adulthood, and (2) deaths of cats by lethal management actions. We further postulated that both types of death can be reduced by taking appropriate management actions (i.e., sterilization to reduce the number of kittens that are born and subjected to potential mortality, and reducing or eliminating lethal management) and therefore collectively defined these as “preventable” deaths. We acknowledge that free-roaming cats sometimes die from outdoor hazards (including predation, vehicles and other accidents, starvation, extreme weather, and lack of medical care) that may be reducible by other kinds of management actions. However, while we included these events in our specification of baseline age-specific mortality rates, our model did not explicitly assign cause of hazard-based death for each individual. Consequently, these deaths were excluded from our definition and calculation of preventable deaths.

We used two approaches to characterize the role of dispersal and abandonment into the focal population. First, the origin (either locally-born or born elsewhere) of each cat in the population over a 10-year period was tabulated within the model. Second, each of the initial 50 cats in the focal population was assigned two unique (but “virtual”) genetic variants (alleles) at a specified locus in *Vortex*, resulting in 100 diagnostic alleles within the starting population. All cats from the neighborhood population were assigned different alleles. Each kitten that was produced from a specific mating pair was assigned one random allele from each parent, permitting the simulated genetic composition of the focal population to be tracked over time.

To investigate the scalability of our results, we repeated our previously published model of sterilization-based management (75% of intact individuals sterilized per time step) over a series of larger starting population sizes (250, 500, 1,000, 2,500, and 5,000 individuals) while holding constant all other parameters used in the original 50-cat model and maintaining the original number of iterations. The number of individuals present in neighborhood populations that served as a reservoir of possible immigrants was also scaled proportionally (with 1,000, 2,000, 4,000, 10,000, and 20,000 respectively, compared to the neighborhood population size of 200 in the original models for the 50-cat focal population). We then examined the resulting population trajectories for different initial population sizes for degree of correspondence.

Finally, we determined the number of cats remaining under each scenario at the end of 10 years, and the origin of these cats. These results allowed us to evaluate tradeoffs and synergies between reducing the number of preventable deaths and reducing population size. More detailed examination of management optimization that also incorporates cost efficiencies will be presented elsewhere.

It is important to note that simulations are approximations of reality, not absolute predictions of future system behavior, and should be interpreted accordingly. However, our model was parameterized using best available empirical information, and we believe that it effectively captures the critical relationships and dynamics of free-roaming cat population function [see (1)]. The numerical output from the model is consistent with expectations based on an array of population studies [e.g., (26, 27, 34, 37–39)]. For example, kitten mortality outcomes and birth rates are in line with empirical data, in addition to making biological sense. Furthermore, there are real-world precedents for all of our tested management scenarios. We are therefore confident that this model provides a robust platform for systematically comparing and contrasting the likely outcomes of different management scenarios.

## Results

**Figure 1:**
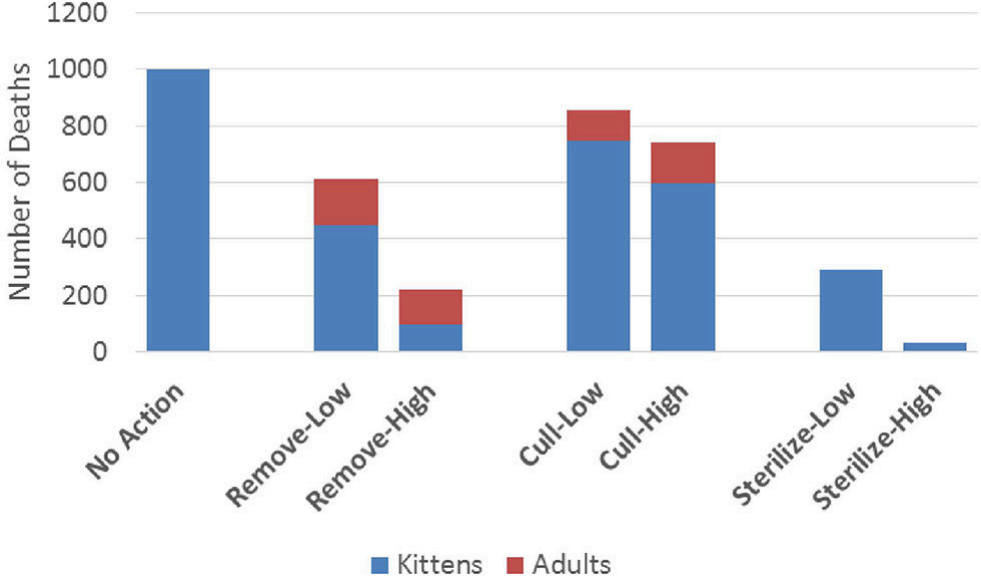
Cumulative number of preventable deaths (kittens that do not survive beyond 6 months of age plus all adults euthanized in “cull” and “remove” scenarios) over a 10-year period for all management scenarios. Parameters of each management scenario are defined in the Methods section.

**Figure 2:**
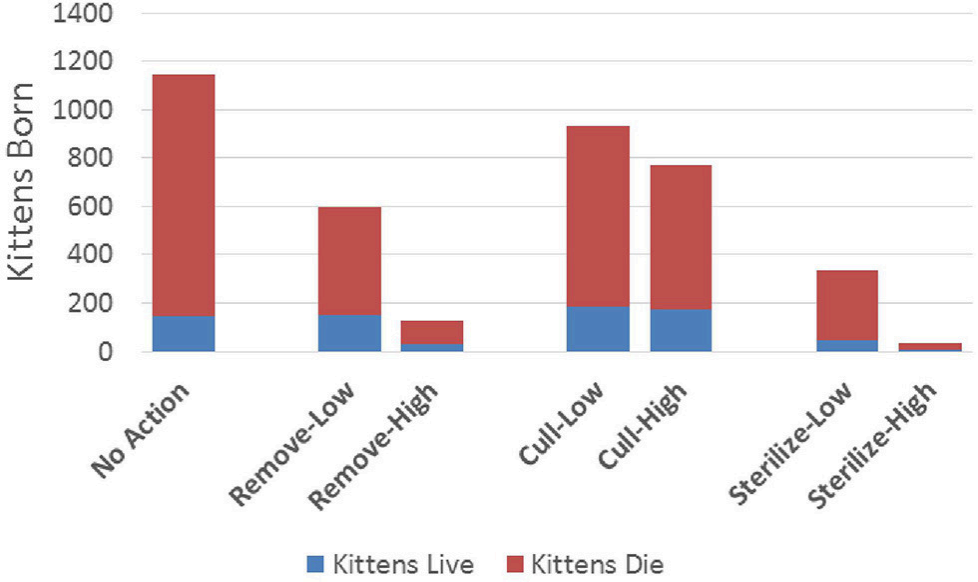
Cumulative number of kittens born in the focal population over 10 years under different management scenarios. “Kittens Live” shows the number that survive beyond six months of age and “Kittens Die” shows the number that die before six months. Parameters of each management scenario are defined in the Methods section.

All management actions that we simulated reduced the number of preventable deaths over 10 years in comparison to taking no action (Figure 1). This reduction was moderate for both of the cull scenarios and for the remove-low scenario, larger for the sterilize-low and remove-high scenarios, and largest for the sterilize-high scenario, which resulted in 31 times fewer preventable deaths than the no action scenario (see Table 2 for detailed quantitative outputs for all scenarios). Preventable deaths were comprised mostly of kittens in all scenarios except remove-high, where it was roughly equal to adult preventable deaths (i.e., lethal removals). The large differences in number of kitten deaths among scenarios was mostly a function of the different number of kittens that were locally-born, as illustrated in Figure 2 (1,146 kittens born locally for no action, 38 for sterilize-high). In contrast, the proportion of all kittens born that survived to 6 months or beyond was relatively small across scenarios, ranging from 20 to 25% for lethal management scenarios and 13 to 16% in all other scenarios (Table 2). This observed difference is consistent with the inclusion of density-dependent kitten survival, where populations that remain near their local carrying capacity will be subject to more stressful conditions and, subsequently, lower survival rates among the youngest age class.

**Table 2:** Scenario-specific outcomes (numbers of cats) from simulation modeling.

	**No action**	**Remove-low**	**Remove-high**	**Cull-low**	**Cull-high**	**Sterilize-low**	**Sterilize-high**
Final population size (Mean)	49.53	26.37	6.80	44.69	39.01	38.74	25.80
SD	3.76	11.38	3.24	5.31	8.90	8.37	7.42
Cumulative number of cats removed or sterilized (Mean)	0	168.02	127.32	109.90	142.55	100.03	103.60
Cumulative preventable deaths, kittens plus adults (Mean)	1000.89	614.91	222.50	858.43	737.98	290.32	32.13
SD	97.63	118.08	37.81	84.15	83.56	62.35	13.00
Min	731	323	144	640	549	154	7
Max	1254	890	347	1111	963	477	75
Cumulative preventable deaths, kittens only (Mean)	1000.89	447.07	95.72	745.97	598.68	290.32	32.13
SD	97.63	114.55	31.52	83.88	78.99	62.35	13.00
Min	731	196	38	543	418	154	7
Max	1254	760	204	980	809	477	75
Cumulative kittens born in focal population (Mean)	1145.86	596.02	127.81	931.91	771.29	336.81	37.80
SD	111.32	156.17	42.84	101.96	96.22	72.93	15.82
Min	860	254	49	688	522	172	11
Max	1404	990	273	1175	1006	544	86
Cumulative kittens surviving to 6 months (Mean)	144.97	148.95	32.09	185.94	172.61	46.49	5.67
SD	33.45	47.31	13.59	36.51	30.80	14.21	4.31
Min	77	47	2	98	93	13	0
Max	263	313	74	282	260	75	16
Cumulative adults ever in focal population (Mean)	241.54	247.97	134.32	279.84	269.04	146.51	107.10
SD	35.19	50.10	17.88	38.57	35.57	17.63	10.62
Min	173	139	91	202	186	96	84
Max	353	432	184	380	365	187	133
Cumulative adults born in focal population (Mean)	188.43	192.95	81.07	228.35	216.06	93.74	54.54
SD	32.37	45.58	13.28	35.87	30.73	13.67	4.09
Min	122	93	51	146	137	62	47
Max	303	350	122	321	301	123	65
Cumulative adults born outside focal population (Mean)	53.11	55.02	53.25	51.49	52.98	52.19	52.56
SD	9.50	11.10	8.72	10.61	11.11	11.08	8.52
Min	25	26	31	25	32	0	33
Max	79	86	74	82	84	82	71
Living adults at 10-year mark born in focal population (Mean)	34.88	18.11	1.05	33.40	28.77	16.49	1.73
SD	5.64	11.14	1.71	6.56	8.40	7.37	2.07
Min	21	0	0	11	0	3	0
Max	48	47	9	48	47	32	10
Living adults at 10-year mark born outside focal population (Mean)	14.52	8.44	4.42	10.45	10.01	22.30	23.86
SD	4.63	3.70	2.72	3.75	3.95	5.47	6.09
Min	5	1	0	4	0	12	13
Max	28	22	15	23	20	37	41

*Sections labeled as “cumulative” were totaled over the 10-year simulation after averaging results across 100 randomly chosen iterations within each time step. Other sections refer to population status at the end of the 10-year simulation, with means based on averaging across all 1,000 iterations. SD, standard deviation; Min, minimum value; Max, maximum value*.

**Figure 3:**
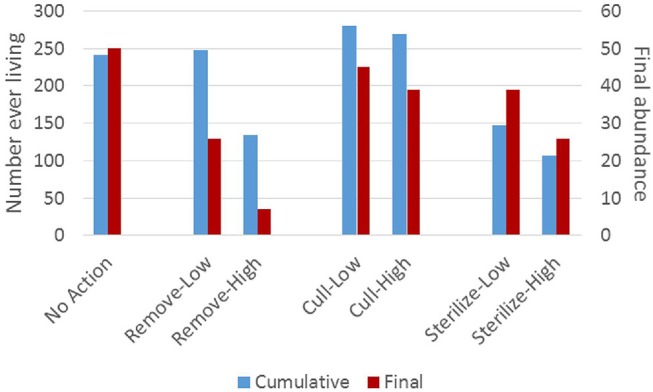
Cumulative number of adult cats (>6 months of age) ever living in the focal population (left-hand vertical axis) and final population size at the 10-year mark (right-hand vertical axis). Parameters of each management scenario are defined in the Methods section.

**Figure 4:**
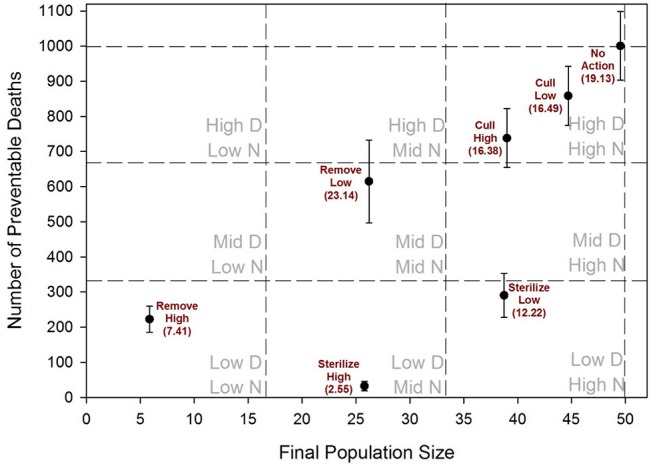
Comparison of management scenarios across a combination of two outcome types; the number of preventable deaths (as defined in Methods) and final population size at the end of 10 years. The squares defined by the dashed lines represent High, Intermediate (Mid), and Low values for premature deaths (=D) and final population size (=N). Error bars give standard deviations across 100 model iterations, with 95%CI values given in parentheses.

The cumulative number of adult cats that ever lived in the focal populations over a 10-year period was slightly increased in both of the cull scenarios in comparison to the no-action scenario, and reduced by about one-half in the remove-high scenario and both sterilize scenarios (Figure 3). Final population size at the 10-year point was reduced only slightly by culling, reduced about one-half by sterilize-high, and reduced the most by remove-high. Remove-low and sterilize-high both resulted in similar ending populations that were about one-half of their original size (Figure 3). However, under remove-low management, a much higher number of cats cumulatively lived in the focal population, and substantially more kittens were born and died than in sterilize-high management. Sterilize-low also had substantially more kitten births and deaths than sterilize-high and resulted in only a modest decline in population size at end of 10 years (Figure 3). Figure 4 provides a graphical summary of each scenario's outcome for cumulative preventable deaths and final population size.

**Figure 5:**
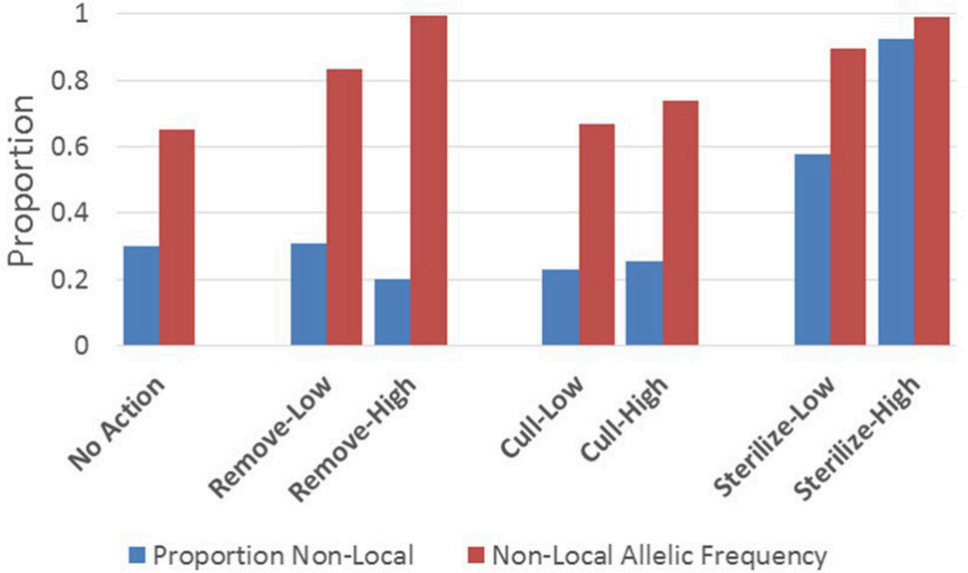
Influx (abandonment and immigration) under all management scenarios indexed by: (1) the proportion of adult cats living at the 10-year mark that were born outside the focal population, and (2) frequency of non-local alleles in the final focal population at the 10-year mark. Parameters of each management scenario are defined in the Methods section.

Although dispersal and abandonment rates into the focal population were fixed within stochastic bounds through the simulations, their cumulative impacts varied substantially across scenarios. At the 10-year point, the proportion of living cats in the focal populations that were born outside the focal population was much higher under the sterilize-high scenario (>90%) than under a no-action scenario (30%) (Figure 5), and little changed by any non-sterilization management option. In partial contrast, influx measured by the frequency of non-local alleles in the focal population at the 10-year mark was higher under remove and sterilize scenarios than under a no-action scenario. The cull scenarios had relatively little effect on allelic frequencies.

**Figure 6:**
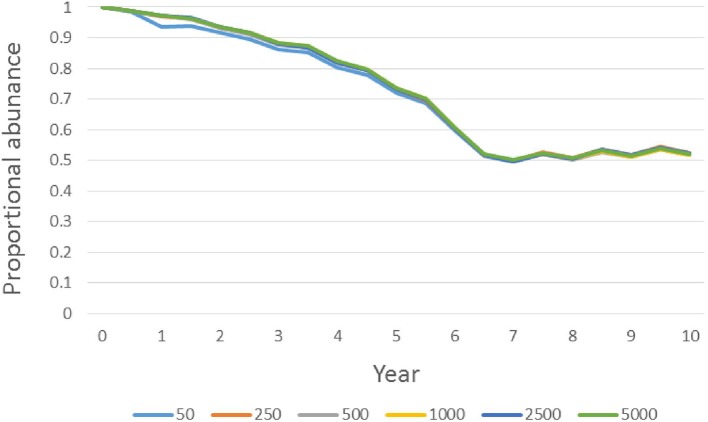
Proportional population size (scaled to initial size) of focal free-roaming cat populations over time across a range of initial population size values for sterilization management where 75% of intact cats are sterilized during each six-month time step and returned to the population (i.e., Sterilize-High; see text for details). The legend indicates the colors of curves associated with different beginning population sizes. Curves for different initial sizes are almost entirely overlapping, so not all are visible.

For the scalability analysis, Figure 6 illustrates that the proportional change in focal population size over a 10-year simulation was very consistent across all tested initial population sizes at the 75% sterilization intensity.

## Discussion

Management of free-roaming cats may have demographic effects that extend across multiple generations and are relevant from a lifesaving perspective. Although we recognize that many factors are not included in our analysis that could affect cat lifespan and quality of life [see (12, 40, 41)], our results suggest that from a cat welfare perspective, we cannot maximize our prospective goals (i.e., saving lives tomorrow) by focusing only on maximizing our proximate goals (i.e., saving lives today). Instead, balancing these goals effectively requires attention to management strategy.

In our judgment the most important findings of this analysis are that:

1. Cumulative preventable deaths, particularly of kittens, over 10 years are much lower for higher-intensity sterilization (TNR) than for all other scenarios.
2. Lower-intensity TNR is comparable to higher-intensity removal in terms of cumulative preventable deaths, but it is less effective at reducing population size.
3. Lack of management (i.e., the no-action scenario) results in more cumulative preventable deaths, particularly of kittens, than any active management option. This includes lethal removal.
4. Under high-intensity TNR, the proportion of cats in the final population that were born elsewhere is the highest of all management options (Figure 5, Table 2). For this reason, reducing abandonment and, where possible, immigration in conjunction with high-intensity TNR could improve outcomes more than for any other management option tested.
5. Culling is likely to be ineffective and inefficient in terms of cumulative preventable deaths and population size reduction.
6. Scalability results suggest that these conclusions apply across a wide range of focal population sizes.

Some of these results may seem counterintuitive, but they are logical consequences of the high reproductive capacity of cats, which can produce many more offspring than are needed to maintain a population at a given carrying capacity (34). Our analysis indicates that as a result of this reproductive capacity, kitten deaths usually comprise a large majority of overall mortalities that can be influenced by management actions or inactions. The animal welfare community has often emphasized preventing deaths from lethal management, but based on these findings may wish to also make reducing kitten deaths an equally explicit management and policy goal. The best management strategy for accomplishing this is to quickly suppress reproduction with high-intensity sterilization, leading to reduced population size over time, and then allow these changes to generate compounded benefits into the future. As a consequence, far fewer kittens will be exposed to intrinsically high mortality rates, and far fewer will die before reaching adulthood.

With sufficient intensity, TNR offers significant advantages in terms of minimizing preventable deaths while also substantially reducing population size. High-intensity TNR programs can be further improved by reducing abandonment, or by combining return to field for some cats with adoption for others [see (15, 36, 42, 43) for examples]. On the other hand, at lower sterilization intensities the longer-term lifesaving advantages of TNR become much less compelling because large numbers of kittens remain subjected to high mortality rates over time.

The choice of management strategy should ideally incorporate multiple factors, including population outcome, cat welfare, cat impacts on wildlife, cost effectiveness, ethics, practicality, tractability, likelihood of success, and political/public support. In addition, it should address local priorities and needs, which can vary substantially. We do not intend to suggest how these factors should be weighed by animal welfare professionals or other policy stakeholders, or to draw conclusions about the relative importance of preventable kitten deaths vs. deaths resulting from lethal management. Rather, we emphasize that management choices are likely to have large, persistent, and indirect effects on preventable mortality that can now be more explicitly considered as a result of this analysis. We further conclude that in the longer-term, the goals of reducing cat population size and minimizing preventable deaths are largely synergistic. Recognizing this potential compatibility may bring the interests of diverse stakeholders into better alignment and facilitate collaborative efforts.

For all these reasons, we believe it is appropriate for the animal welfare community to explicitly consider these broader perspectives in developing their goals and strategies for outdoor cat policy and management, and to recognize that TNR intensity is critically important not only to reduce population size, but also to minimize preventable deaths of kittens. We also emphasize the value of collecting standardized monitoring data in support of TNR programs to refine model-based guidance and to improve our understanding of best practices (18). Currently, some TNR practitioners are promoting the concept of “targeting” and focus of resources in locations of highest value for cat population management, which could lead to higher-intensity TNR implementations^3^ (accessed October 27, 2018) (6). These concepts, along with use of appropriate tools and protocols to measure progress and outcomes (18, 44), should be further explored and evaluated as potential “best practices.”

## Author Contributions

PM constructed and implemented simulation models. PM and JDB analyzed and summarized data. PM, JDB, VB, JRB, DL, MS, JL, and SZ contributed to simulation model design and construction. JDB, PM, JRB, and VB conceived and designed this study and wrote this report. DL, MS, JL, and SZ reviewed and edited this report.

### Conflict of Interest Statement

The authors declare that the research was conducted in the absence of any commercial or financial relationships that could be construed as a potential conflict of interest.

## Footnotes

^1^http://shelteranimalscount.org/docs/default-source/DataResources/sac_basicdatamatrix.pdf?sfvrsn=2 (accessed October 16, 2018).

^2^https://www.maddiesfund.org/lifesaving.htm (accessed October 16, 2018).

^3^http://support.petsmartcharities.org/site/DocServer/Targeted_TNR_RW_Summit_v3.pdf?docID=1402
