# Supplementary material for: A Long-Term Lens: Cumulative Impacts of Free-Roaming Cat Management Strategy and Intensity on Preventable Cat Mortalities
Source: Front Vet Sci. 2019 Jul 26;6:238. doi: 10.3389/fvets.2019.00238 (PMC6676151; doi:10.3389/fvets.2019.00238)
Supplement: Supplementary file 1 [file Data_Sheet_1.docx]

A Long-Term Lens: Cumulative Impacts of Free-Roaming Cat Management Strategy and Intensity on Preventable Cat Mortalities

John D. Boone^1*^, Philip S. Miller^2^, Joyce R. Briggs^3^, Valerie A. W. Benka^3^, Dennis F. Lawler^4^, Margaret Slater^5^, Julie K. Levy^6^, and Stephen Zawistowski^7^

^1^Great Basin Bird Observatory, Reno, Nevada, United States of America

^2^Conservation Planning Specialist Group, Species Survival Commission, International Union for Conservation of Nature, Apple Valley, Minnesota, United States of America

^3^Alliance for Contraception in Cats and Dogs, Portland, Oregon, United States of America

^4^Illinois State Museum, Springfield, Illinois, United States of America

^5^Strategy and Research Department, American Society for the Prevention of Cruelty to Animals, Florence, Massachusetts, United States of America

^6^Maddie’s Shelter Medicine Program, Department of Small Animal Clinical Sciences, University of Florida, Gainesville, Florida, United States of America

^7^Animal Behavior and Conservation Program, Hunter College, New York New York, United States of America

*** Correspondence:**Dr. John D. Boone
[boone@gbbo.org](mailto:boone@gbbo.org)

**Supporting Information**

Baseline Input Parameters for Demographic Simulation Models

Data for use as input to our simulation models were obtained from the peer-reviewed literature where available and appropriate. We created a database of literature and, subsequently, a matrix of demographic values, corresponding to specific *Vortex* input fields where available (this matrix is available on the ACC&D website). Where specific data were not available in the scientific literature, we used expert judgment to derive input parameter values through a process of consensus. More detailed information on the underlying structure, algorithms and approach used in *Vortex* is available in Lacy et al. (2018).

Timestep for all simulations: The timestep for this type of demographic analysis defines the interval over which demographic rates are applied to individuals within the population, and over which summary population statistics are calculated. Because free-roaming cats tend to have a more seasonal pattern of reproduction, and early onset of reproductive activity, we used a timestep for our simulations of six months. On a calendar basis, these timesteps would roughly correspond to a “spring/summer” timestep featuring high reproductive rates, and an “autumn/winter” timestep with correspondingly lower reproductive rates. All reproductive and survival probabilities are therefore calculated on the basis of this timestep.

Metapopulation structure: We employed a simplified metapopulation structure in all model scenarios. This means we set up two subpopulations that together comprised the metapopulation: the focal population to which all of our treatment scenarios were directed, and the “neighborhood” to which that focal population was demographically connected through occasional dispersal of individual animals. This configuration required us to specify demographic input parameters for the neighborhood population as well, even if we are not interested in tracking the long-term dynamics of that population. We assumed that the neighborhood population has the same basic demographic structure as our focal population, in other words, equivalent rates of reproduction and survival. The only difference is that the neighborhood population is four times larger than the focal population in both initial population size and carrying capacity (see below).

Dispersal in our metapopulation scenarios must be characterized in some detail. Although many studies document the presence of immigration and emigration among local cat populations (e.g., Castillo and Clarke 2003; Levy et al. 2008; Centonze and Levy 2002; Jöchle and Jöchle 1993), the specific demographic characteristics of those dispersing individuals are not similarly available from the literature. Therefore, we assumed that dispersing individuals are between 6 and 24 months of age, with males making up approximately 75% of the dispersing individuals. We assumed a rate of dispersal between focal and neighborhood populations of 2% per timestep. This means that, on average, a population of 200 individuals will have one female and three males age 6-24 months randomly chosen to disperse to the other population in a given timestep. Although these rates of dispersal between focal and neighborhood populations are the same, the 4-fold larger size of the surrounding neighborhood population means that the number of individuals entering the focal population was considerably larger than the number leaving that population. Finally, we also assumed there is a cost to dispersal across human-dominated urban landscapes, expressed as a 75% survival rate among those individuals designated as dispersers in any given timestep.

Litter abandonment: Our metapopulation model structure also included the abandonment of unwanted litters from owned-cat households. We implemented this through the same Dispersal module in *Vortex* as described above. We assumed that approximately one “litter equivalent” of 2-3 6-month-old animals is added to the focal population each 6-month timestep, with an expected equal sex ratio. We used the concept of “litter equivalent” to represent one abandoned litter with 100% survival to the next timestep, or two abandoned litters with 50% survival (perhaps abandoned at a younger age and subject to higher mortality before the next timestep), etc. to create the desired number of animals that were added to the focal population.

Breeding system: Free-roaming cats display a classic polygynandrous breeding system, where both males and females may mate with multiple partners. Individual litters with multiple paternity have been observed, but this is not important from a population demographic standpoint as we are not explicitly including population-level genetic considerations in our analyses, and we assumed that the size of litters sired by multiple males will be the same as those sired by a single male. Therefore, we opted for a standard polygynous breeding system for our model structure.

Age of first reproduction: Free-roaming cats become reproductive at 6-12 months of age (median age 10.5 months, interquartile range 8 – 12 months (Nutter 2005). For our purposes, we set the age of first reproduction at 6 months, meaning that an individual reaching 6 months of age will have an opportunity to breed once before aging to the next timestep (i.e., before becoming 12 months old).

Maximum age of reproduction: In its simplest form, *Vortex* assumes that animals can reproduce (at the normal rate) throughout their adult life. Under this assumption, individuals can reproduce until they die, meaning that there is no reproductive senescence in our model. Free-roaming cats are clearly capable of breeding successfully for many years if they escape mortality from natural or anthropogenic causes. There is considerable variance around estimates of typical free-roaming cat longevity. Many sources suggest a typical lifespan of approximately 3 – 5 years of age (Anderson et al., 2004; Foley et al., 2005). We assumed for our models a maximum possible age of 6.5 years (i.e., 13 timesteps). In other words, an animal that reaches 6.5 years of age – 13 time steps – is allowed to go through one final breeding cycle before being removed from the population through additional mortality. Note that the probability of a newborn kitten actually reaching this age is no more than about 10%. This maximum age may be an optimistic estimate, but we evaluate reproductive management options while effectively holding this longevity parameter value constant across all scenarios.

Kitten production: For all models discussed here, we defined reproduction as the successful live birth of a litter of kittens, before weaning has taken place. An extensive dataset (Nutter 2005) showed litter production at an average rate of 1.4 litters per female per year, demonstrating the ability of adult females to produce more than one litter in a year. Furthermore, their data suggested a strong seasonality to litter production. We specified in *Vortex* that an adult female will produce no more than one litter in any one timestep. Assuming a rough 2:1 ratio of litters produced in the “spring/summer” timestep compared to “autumn/winter”, and given the overall mean litter production per year, we then assumed that on average 92% of free-roaming adult females produced a single litter in the more favorable timestep, and 48% produced a litter in the less favorable timestep (Figure S1). We recognized that, in theory, a given female could actually produce both litters in the same 6-month interval, thereby failing to reproduce in the next interval. The structure of our model did not allow for this event, but we were confident that the overall demographic result of our model structure is very similar to that which may occur in nature.

We allowed the breeding rate to vary randomly from one breeding cycle to the next to account for random environmental factors influencing mean rates. This source of variability is often referred to as environmental stochasticity, and is defined operationally as a standard deviation applied to the mean rates at each timestep. As in the case of the mean rates, we specified a seasonal form of environmental variability, with a standard deviation in the “spring/summer” timestep of 3% and 12% in the “autumn/winter” timestep. We assumed that reproduction in the lower-productivity season will be more highly variable than the more optimal season.

Data on litter size (Nutter 2005) indicated a median of 3 kittens/litter, with a range of 1 to 6. Note that this median value does not include stillbirths and/or aborted fetuses, but instead specifies live births. We agreed on a mean distribution of 3.47 kittens/litter, with the specific distribution as described in Table S1. Across all litters born in a given timestep, we assume a 50:50 sex ratio.

Density dependence in reproduction: The inclusion of density dependence is an important consideration in demographic modeling efforts such as this. We chose a strategy where high density does not reduce the proportion of females that produce litters, but instead reduces the survivorship of those kittens after they are born. For more details, see the discussion on mortality rates below.

Male breeding rates: In some species, a proportion of adult males may be socially restricted from breeding despite being physiologically capable. This can be modeled in *Vortex* by specifying a portion of the total pool of adult males that may be considered “available” for breeding each timestep. We do not have detailed data on this parameter, so we assumed that all reproductively intact adult males are available for breeding each timestep. This is most probably a reasonable assumption, as population dynamics of polygynous species such as the domestic cat are very rarely male-limited. In the model structure employed by *Vortex*, males in a polygynous breeding system will not be limiting, as individuals may be used multiple times for mating with the group of available females.

Mortality rates: *Vortex* defines mortality as the annual rate of age-specific death from timestep *x* to *x* + 1; in the language of life-table analysis, this is equivalent to *q*(*x*). We assigned a mean value of 75% mortality for kittens from live birth to six months of age (Nutter 2005), with an assigned standard deviation around that mean due to environmental variability of 10%. While there are other estimates of lower kitten mortality (e.g., Centonze and Levy 2002), we believed that the study cited above provides a robust estimate of this parameter. If an adult female produces two litters in a given year, we assumed equal mortality across both litters, in accord with a previous study (Nutter 2005) that observed no significant influence of litter order on mortality. We recognized, however, that high-density cat populations will likely experience higher levels of kitten mortality as food and other resources become more limiting. We assumed that kitten mortality would increase to a value of 90% when the population abundance approached the local habitat carrying capacity, *K* (see below for more information on this $Mort_{Kit}=100-\left[S_{0}-\left(\left(S_{0}-S_{K}\right)\left(\frac{N}{K}\right)^{6}\right)\right]$parameter). The specific functional form of this relationship is:

where *S*_0_ (25%) is the survival at low density, *S_K_* (10%) is the survival rate at high density, *N* is population size, and *K* is carrying capacity (Figure S2).

Estimates of annual adult survival rates vary widely across the literature, from just under 0.9 in an urban population (Schmidt et al. 2009) to 0.55 – 0.78 in a rural population (Jöchle and Jöchle 1993). It may not be surprising to see higher adult survival in urban areas where supplemental feeding may be frequent, and overall resource availability may be enhanced. An urban annual survival rate around 90% appears commonly in the literature, so we agreed to set this as our baseline rate for our simulated populations. This equated to a survival rate of 0.948 per 6-month timestep, or a mortality rate (in the language of *Vortex*) of 0.052 per timestep.

We did not explore the impact of geographic location of free-roaming cat populations and the resulting impact on demography. For example, we considered that populations in higher latitudes would potentially experience greater seasonal variation in reproduction and/or mortality. Future versions of the model structure described here could be adapted to include this factor.

Catastrophes: Catastrophes are unusual environmental events that are outside the bounds of normal environmental variation affecting reproduction and/or survival. Natural catastrophes can be tornadoes, floods, droughts, disease, or similar events. These events are modeled in *Vortex* by assigning an annual probability of occurrence and a pair of severity factors describing their impact on mortality (across all age-sex classes) and on the proportion of females successfully breeding in a given year.

We agreed to exclude catastrophic events from our current set of models for purposes of simplicity. The mechanisms and impacts of such events on free-roaming cat populations, particularly those inhabiting highly modified environments in urban landscapes, are not well understood at this point. Future modeling efforts may be conducted to explicitly investigate the sensitivity of model outcome to the addition of catastrophes.

Inbreeding depression: *Vortex* provides the option to model the detrimental effects of inbreeding, most directly through reduced survival of offspring through their first 6-12 months. Detailed data on the presence and intensity of inbreeding and its impacts on free-roaming cat populations are not available. The impact of past inbreeding history, and the social structure of free-roaming cat populations, makes an estimate of inbreeding depression extent and severity highly speculative at best. Observed morphological diversity among free-roaming cats suggests that they possess at least moderate underlying genetic heterogeneity. Therefore, we excluded inbreeding depression as an additional factor in our models.

Initial population size and carrying capacity: All models were initialized with a starting population abundance of 50 cats in the focal population and 200 cats in the surrounding neighborhood. All individuals of each sex were sorted into appropriate age classes according to the stable age distribution that is calculated from the rates of reproduction and survival used as model input. This distribution is shown in Table S2.

The ecological carrying capacity, *K*, for a given habitat patch defined an upper limit for the population size, above which additional mortality was imposed randomly across all age classes in order to return the population to the value set for *K*. In all models constructed for this report, both the focal and neighborhood populations begin the simulations with an initial abundance equivalent to the carrying capacity for that habitat. This is a reasonable starting condition for analysis of reproductive management of free-roaming cat populations, as management issues will become highest priority when cat populations reach their maximum density and begin to generate higher levels of disease transmission risk, nuisance behaviors, and the like. Carrying capacity values for each population type were estimated by assigning a density to each type, and extrapolating that value through a spatial extent of approximately 12.5Ha = 0.125km^2^. This method assumed, of course, that the assigned density represented something close to a maximum value that could most accurately reflect a meaningful carrying capacity estimate. The density values used for this analysis were derived from a summary (Turner and Bateson 2000) citing multiple original data sources.

Iterations and years of projection: Our stochastic simulation model features random variation in demographic parameters, yielding different results for different runs. Therefore, we generated multiple replicates to assess overall simulated population behavior. All scenarios were simulated 1000 times, with each projection extending to 20 timesteps (equivalent to 10 years).

Model Structures to Simulate Population Management Options

All scenarios featured a third population, known as the Shelter, to which trapped individuals were moved for treatment. Cats were moved to the Shelter using the Harvest module in *Vortex*, with individuals chosen at random from the focal population to simulate the trapping process and then assigned residency in the Shelter for treatment. All trapping was assumed to take place before the onset of discrete breeding seasons, thereby limiting kitten production to those that were not trapped in a given timestep. Males and females were assumed to be trapped with equal probability.

Note that only individuals in our focal population were trapped and subject to treatment. In the case when cats were sterilized, they were returned there and were then allowed to disperse to the neighborhood population and remain reproductively inactive. Similarly, individuals in the neighborhood population could randomly disperse to the focal population and therefore be susceptible to trapping and treatment.

*Remove*: Cats were randomly chosen (trapped) from the focal population and moved to the Shelter. We set the breeding rates within the Shelter at 0%, prohibiting kitten production from taking place. Additionally, we set the mortality rates in this location to be 100%, thereby clearing out the Shelter population by the end of each timestep. The actual fate of individual cats subject to the Removal strategy is not an explicit part of the demographic model; the key feature here is that cats subject to a Removal strategy were not returned to the focal population.

We tested average removal rates in the focal population of 25% (Low) and 50% (High) of the standing population, calculated at each 6-month timestep. The actual number of cats removed was subject to stochastic sampling variation at each timestep; therefore, a Population State Variable (PSVar) called *Number Treated* was created within Vortex to track the actual number of individuals trapped. The properties of this PSVar are described below.

*Sterilize*: As with Removal scenarios, cats were randomly chosen (trapped) from the focal population and moved to the Shelter population for treatment. We created the Individual State Variable (ISVar) *Treated* that defined the treatment status – equivalently, the reproduction state – of any individual in the focal population. *Treated* = 0 means an individual was not trapped and treated and therefore reproductively active, while *Treated* = 1 means they were reproductively inactive, i.e., sterilized. Within any given sterilization scenario, all individuals began the simulation as active (in the language of *Vortex*, Initialization function = 0), and all individuals born during the simulation started their lives reproductively active (i.e., Birth function = 0). An individual would “transition” from the active to the inactive state if trapped according to a specified probability. For example, if the goal was to trap 50% of the total breeding (previously untreated) population, the *Vortex* transition function for each qualifying individual would flip an individual from *Treated* = 0 to *Treated* = 1 if a random number drawn for that individual was less than or equal to the probability of being trapped, in this case, 0.5. Importantly, in this and other scenarios described below, we simulated a system where individuals were trapped and brought to the Shelter for treatment only if they had not been treated previously (i.e., *Treated* = 0). Logically, then, we assumed over the time period of the simulation that a larger proportion of the total population would have to be trapped to achieve a given target treatment rate. The process of trapping was not explicitly modeled here; only the proportion of individuals treated was explicitly considered.

Once an intact individual was trapped and treated, its probability of breeding was taken to be 0. A reproductively active female would breed at the baseline rate specified in the previous section. If a male was trapped and sterilized, it is considered no longer a member of the “pool” of available mates, so the total available pool was adjusted accordingly, proportional to the total number of sterilized males.

We tested sterilization rates equal to 25% (Low) and 75% (High) of the untreated population, calculated at each 6-month timestep. Males and females were assumed to be trapped with equal probability. These rates were applied each timestep of the simulation, with additional State Variables created to tally the total number of individuals within the focal population that were either fertile (untreated) or treated (sterile). These were used to estimate trapping intensities required to successfully trap and treat the desired proportion of the target (fertile) population at each timestep.

*Vortex* Syntax for State Variable Descriptions and Demographic Impacts

Below is a listing of the specific equations describing the set of Individual State Variables (ISVs) used to simulate the four population management options, and the equations used to describe their impacts on free-roaming cat reproduction or mortality. The specific elements of Individual State Variable characterization are:

Initialization Function (Init fn): The value of that ISV assigned to all individuals making up the initial population at the beginning of the simulation.

Birth Function (Birth fn): The value of that ISV assigned to all individuals born into the population at each timestep.

Transition Function (Trans fn): The mathematical formulation describing the change in ISV value from one timestep to the next.

Variables used in the ISV functions:

*A* Age

*P* Population number, a numerical identifier for each subpopulation in a metapopulation (used to restrict treatment to focal population (P = 1))

Management Strategy: Removal

| PSVar | Label | Init fn | Trans fn |
| --- | --- | --- | --- |
| PS1 | Number Treated | 0 | =HARVESTS |

Female breeding rate = (Normal breeding rate)

= 48+(44*(Y%2=1))[Modulus operator (Y%2=1) controls seasonal rates]

Management Strategy: Sterilize

| SV | Label | Init fn | Birth fn | Trans fn |
| --- | --- | --- | --- | --- |
| IS1 | Treated | 0 | 0 | =(P=3)+IS1 |
| IS2 | FertileFocalPop | 0 | 0 | =(P=1)*(IS1=0) |
| IS3 | SterileFocalPop | 0 | 0 | =(P=1)*(IS1>0) |
| PS1 | Number Treated | 0 |  | =HARVESTS |

Female breeding rate = (ReproState=0)(Normal breeding rate)

=(IS1=0)*(48+(44*(Y%2=1))[Modulus operator(Y%2=1) controls seasonal rates]

Below are specific examples of detailed, scenario-specific summaries for a full accounting of the input data required for the *Vortex* models used in this analysis.

VORTEX 10.3.5.0 -- simulation of population dynamics

Project: FinalAnalysis_Jan2018

**Scenario: Removal_25**

3 populations simulated for 20 years for 1000 iterations

Sequence of events in each time cycle:

EV

Harvest

ISUpdate

PSUpdate

Supplement

Breed

Mortality

Age

Disperse

rCalc

Ktruncation

ISUpdate

GSUpdate

Census

Extinction defined as no males or no females.

No inbreeding depression.

Populations:

Focal Pop

Population state variables

PS1: Kittens Initialization: 0 Transition: =PS1

PS2: Adults Initialization: 0 Transition: =PS2

PS3: Number Treated Initialization: 0 Transition: =HARVESTS

Neighborhood

Population state variables

PS1: Kittens Initialization: 0 Transition: =PS1

PS2: Adults Initialization: 0 Transition: =PS2

PS3: Number Treated Initialization: 0 Transition: =PS3

Shelter

Population state variables

PS1: Kittens Initialization: 0 Transition: =ITOT1

PS2: Adults Initialization: 0 Transition: =ITOT2

PS3: Number Treated Initialization: 0 Transition: =PS3

Individual state variables

IS1: Kitten Initialization: 0 Birth function: 0 Transition: =(A=1)

IS2: Adult Initialization: 0 Birth function: 0 Transition: =(A>1)

Correlation of EV among populations = 0.5

Both sexes disperse, from age 1 to age 4

Survival during dispersal: 75

Dispersal rates (as percents), from source (row) to destination (column):

Focal Pop Neighborhood Shelter

Focal Pop =4*(0.25+(0.5*(S='M'))) 0

Neighborhood =(4*(0.25+(0.5*(S='M'))))+((A=1)*5)) 0

Shelter 0 0

Reproductive System:

Polygyny, with new selection of mates each year

Females breed from age 1 to age 13

Males breed from age 1 to age 13

Maximum age of survival: 13

Sex ratio (percent males) at birth: 50

Correlation of EV between reproduction and survival = 1

EV sampled from binomial distributions.

Population specific rates for Focal Pop

Percent of adult females breeding each year: =48+(44*(Y%2=1)) with EV(SD): =3+(12*(Y%2=1))

Percent of adult males in the pool of breeders: 100

Brood size distribution:

3 percent size 1

5 percent size 2

50 percent size 3

30 percent size 4

8 percent size 5

4 percent size 6

Female annual mortality rates (as percents):

Age 0 to 1: =100-(25-((25-10)*((N/K)^6) with EV(SD): 10

After age 1: 5.2 with EV(SD): 2

Male annual mortality rates (as percents):

Age 0 to 1: =100-(25-((25-10)*((N/K)^6) with EV(SD): 10

After age 1: 5.2 with EV(SD): 2

Initial population size:

Age 0 1 2 3 4 5 6 7 8 9 10 11 12 13 Total

Females 0 6 5 3 3 2 2 1 1 1 0 0 1 0 25

Males 0 6 5 3 3 2 2 1 1 1 0 0 1 0 25

Carrying capacity: 50 with EV(SD): 0

Harvest from year 1 through year 20 by increments of 1 of individuals that =RAND<0.25

Age 0 1

Females 0 50

Males 0 50

Supplementation from year 1 through year 100 by increments of 1

Age 0 1

Females 0 0

Males 0 0

Population specific rates for Neighborhood

Percent of adult females breeding each year: =48+(44*(Y%2=1))

with EV(SD): =3+(12*(Y%2=1))

Percent of adult males in the pool of breeders: 100

Brood size distribution:

3 percent size 1

5 percent size 2

50 percent size 3

30 percent size 4

8 percent size 5

4 percent size 6

Female annual mortality rates (as percents):

Age 0 to 1: =100-(25-((25-10)*((N/K)^6) with EV(SD): 10

After age 1: 5.2 with EV(SD): 2

Male annual mortality rates (as percents):

Age 0 to 1: =100-(25-((25-10)*((N/K)^6) with EV(SD): 10

After age 1: 5.2 with EV(SD): 2

Initial population size:

Age 0 1 2 3 4 5 6 7 8 9 10 11 12 13 Total

Females 0 30 21 14 12 7 5 3 3 2 2 1 0 0 100

Males 0 30 21 14 12 7 5 3 3 2 2 1 0 0 100

Carrying capacity: 200 with EV(SD): 0

Population specific rates for Shelter

Percent of adult females breeding each year: 0 with EV(SD): 0

Percent of adult males in the pool of breeders: 100

Brood size distribution:

0 percent size 1

0 percent size 2

0 percent size 3

0 percent size 4

0 percent size 5

100 percent size 6

Female annual mortality rates (as percents):

Age 0 to 1: 100 with EV(SD): 0

After age 1: 100 with EV(SD): 0

Male annual mortality rates (as percents):

Age 0 to 1: 100 with EV(SD): 0

After age 1: 100 with EV(SD): 0

Initial population size:

Age 0 1 2 3 4 5 6 7 8 9 10 11 12 13 Total

Females 0 0 0 0 0 0 0 0 0 0 0 0 0 0 0

Males 0 0 0 0 0 0 0 0 0 0 0 0 0 0 0

Carrying capacity: 200 with EV(SD): 0

Translocation via population 3

with % survival during harvest: 100

and % survival during supplementation: 100

VORTEX 10.3.5.0 -- simulation of population dynamics

Project: FinalAnalysis_Jan2018

**Scenario: Sterilize_75**

3 populations simulated for 20 years for 1000 iterations

Sequence of events in each time cycle:

EV

Harvest

ISUpdate

PSUpdate

Supplement

Breed

Mortality

Age

Disperse

rCalc

Ktruncation

ISUpdate

GSUpdate

Census

Extinction defined as no males or no females.

No inbreeding depression.

Global state variables

GS1: TreatTarget_FocalPop Initialization: 0 Transition: =ITOT4

GS2: Fertile_FocalPop Initialization: 0 Transition: =ITOT5

GS3: Sterile_FocalPop Initialization: 0 Transition: =ITOT6

GS4: PropAbundance Initialization: 1 Transition: =(NN1)/50

Populations:

Focal Pop

Population state variables

PS1: Kittens Initialization: 0 Transition: =PS1

PS2: Adults Initialization: 0 Transition: =PS2

PS3: Number Treated Initialization: 0 Transition: =HARVESTS

Neighborhood

Population state variables

PS1: Kittens Initialization: 0 Transition: =PS1

PS2: Adults Initialization: 0 Transition: =PS2

PS3: Number Treated Initialization: 0 Transition: =PS3

Shelter

Population state variables

PS1: Kittens Initialization: 0 Transition: =ITOT2

PS2: Adults Initialization: 0 Transition: =ITOT3

PS3: Number Treated Initialization: 0 Transition: =PS3

Individual state variables

IS1: Treated Initialization: 0 Birth function: 0 Transition: =(P=3)+IS1

IS2: Kitten Initialization: 0 Birth function: 0 Transition: =(A=1)

IS3: Adult Initialization: 0 Birth function: 0 Transition: =(A>1)

IS4: TargetFocalPop Initialization: 0 Birth function: 0 Transition: =(P=1)*(IS1=0)

IS5: FertileFocalPop Initialization: 0 Birth function: 0 Transition: =(P=1)*(IS1=0)

IS6: SterileFocalPo Initialization: 0 Birth function: 0 Transition: =(P=1)*(IS1>0)

Correlation of EV among populations = 0.5

Both sexes disperse, from age 1 to age 4

Survival during dispersal: 75

Dispersal rates (as percents), from source (row) to destination (column):

Focal Pop Neighborhood Shelter

Focal Pop =4*(0.25+(0.5*(S='M'))) 0

Neighborhood =(4*(0.25+(0.5*(S='M'))))+((A=1)*5)) 0

Shelter 0 0

Reproductive System:

Polygyny, with new selection of mates each year

Females breed from age 1 to age 13

Males breed from age 1 to age 13

Maximum age of survival: 13

Sex ratio (percent males) at birth: 50

Correlation of EV between reproduction and survival = 1

EV sampled from binomial distributions.

Population specific rates for Focal Pop

Percent of adult females breeding each year: =(IS1=0)*(48+(44*(Y%2=1)))

with EV(SD): =3+(12*(Y%2=1))

Percent of adult males in the pool of breeders: 100

Brood size distribution:

3 percent size 1

5 percent size 2

50 percent size 3

30 percent size 4

8 percent size 5

4 percent size 6

Female annual mortality rates (as percents):

Age 0 to 1: =100-(25-((25-10)*((N/K)^6) with EV(SD): 10

After age 1: 5.2 with EV(SD): 2

Male annual mortality rates (as percents):

Age 0 to 1: =100-(25-((25-10)*((N/K)^6) with EV(SD): 10

After age 1: 5.2 with EV(SD): 2

Initial population size:

Age 0 1 2 3 4 5 6 7 8 9 10 11 12 13 Total

Females 0 6 5 3 3 2 2 1 1 1 0 0 1 0 25

Males 0 6 5 3 3 2 2 1 1 1 0 0 1 0 25

Carrying capacity: 50 with EV(SD): 0

Harvest from year 1 through year 20 by increments of 1

of individuals that =(IS1=0)*(RAND<0.75)

Age 0 1

Females 0 70

Males 0 70

Supplementation from year 1 through year 100 by increments of 1

Age 0 1

Females 0 70

Males 0 70

Population specific rates for Neighborhood

Percent of adult females breeding each year: =(IS1=0)*(48+(44*(Y%2=1)))

with EV(SD): =3+(12*(Y%2=1))

Percent of adult males in the pool of breeders: 100

Brood size distribution:

3 percent size 1

5 percent size 2

50 percent size 3

30 percent size 4

8 percent size 5

4 percent size 6

Female annual mortality rates (as percents):

Age 0 to 1: =100-(25-((25-10)*((N/K)^6) with EV(SD): 10

After age 1: 5.2 with EV(SD): 2

Male annual mortality rates (as percents):

Age 0 to 1: =100-(25-((25-10)*((N/K)^6) with EV(SD): 10

After age 1: 5.2 with EV(SD): 2

Initial population size:

Age 0 1 2 3 4 5 6 7 8 9 10 11 12 13 Total

Females 0 30 21 14 12 7 5 3 3 2 2 1 0 0 100

Males 0 30 21 14 12 7 5 3 3 2 2 1 0 0 100

Carrying capacity: 200 with EV(SD): 0

Population specific rates for Shelter

Percent of adult females breeding each year: 0

with EV(SD): 0

Percent of adult males in the pool of breeders: 100

Brood size distribution:

0 percent size 1

0 percent size 2

0 percent size 3

0 percent size 4

0 percent size 5

100 percent size 6

Female annual mortality rates (as percents):

Age 0 to 1: 100 with EV(SD): 0

After age 1: 100 with EV(SD): 0

Male annual mortality rates (as percents):

Age 0 to 1: 100 with EV(SD): 0

After age 1: 100 with EV(SD): 0

Initial population size:

Age 0 1 2 3 4 5 6 7 8 9 10 11 12 13 Total

Females 0 0 0 0 0 0 0 0 0 0 0 0 0 0 0

Males 0 0 0 0 0 0 0 0 0 0 0 0 0 0 0

Carrying capacity: 200 with EV(SD): 0

Translocation via population 3

with % survival during harvest: 100

and % survival during supplementation: 100

Translocation criteria for releasing animals: =IS1>0

**Supporting References**

Andersen, M.C., Martin, B.J., and Roemer, G.W. 2004. Use of matrix population models to estimate the efficacy of euthanasia versus trap-neuter-return for management of free-roaming cats. Journal of the American Veterinary Medical Association 225: 1871-1876.

Castillo, D., and Clarke, A.L. 2003. Trap-Neuter-Release methods ineffective in controlling domestic cat “colonies” on public lands. Natural Areas Journal 23: 247-253.

Centonze, L.A., and Levy, J.K. 2002. Characteristics of free-roaming cats and their caretakers. Journal of the American Veterinary Medical Association 220: 1627-1633.

Foley, P., Foley, J.E., Levy, J.K., and Paik, T. 2005. Analysis of the impact of trap-neuter-return programs on populations of feral cats. Journal of the American Veterinary Medical Association 227: 1775-1781.

Jöchle, W., and Jöchle, M. 1993. Reproduction in a feral cat population and its control with a prolactin inhibitor, cabergoline. Journal of Reproduction and Fertility 47(Suppl): 419-424.

Lacy, R.C., Miller, P.S., and Traylor-Holzer, K. 2018. *Vortex 10 User’s Manual*. 1 June 2018 Update. IUCN SSC Conservation Planning Specialist Group, and Chicago Zoological Society, Apple Valley, Minnesota, USA.

Levy, J.K., Gale, D.W., and Gale, L.A. 2008. Evaluation of the effect of a long-term trap-neuter-return and adoption program on a free-roaming cat population. Journal of the American Veterinary Medical Association 222: 42-46.

Nutter, F.B. 2005. Evaluation of a trap-neuter-return management program for feral cat colonies: Population dynamics, home ranges, and potentially zoonotic diseases. Dissertation, North Carolina State University.

Schmidt, P.M., Swannack, T.M., Lopez, R.R., and Slater, M.R. 2009. Evaluation of euthanasia and trap-neuter-return (TNR) programs in managing free-roaming cat populations. Wildlife Research 36: 117-125.

Turner, D.C., and Bateson, P. 2000. *The Domestic Cat: The Biology of its Behaviour*. Cambridge, England: Cambridge University Press. 244 p.

**Table S-1.** Average distribution of litter size across all breeding females in the focal and neighborhood populations as specified in the basic *Vortex* demographic simulation model structure.

| Number of kittens | Percentage of all litters |
| --- | --- |
| 1 | 3 |
| 2 | 5 |
| 3 | 50 |
| 4 | 30 |
| 5 | 8 |
| 6 | 4 |

**Table S-2.** Initial age-specific abundances for the focal and neighborhood populations as specified in the basic *Vortex* demographic simulation model structure. Abundance values are split evenly among males and females within each age class.

| Age (6-month interval) | Focal Population | Neighborhood Population |
| --- | --- | --- |
| 1 | 12 | 60 |
| 2 | 10 | 42 |
| 3 | 6 | 28 |
| 4 | 6 | 24 |
| 5 | 4 | 14 |
| 6 | 4 | 10 |
| 7 | 2 | 6 |
| 8 | 2 | 6 |
| 9 | 2 | 4 |
| 10 | 0 | 4 |
| 11 | 0 | 2 |
| 12 | 2 | 0 |
| 13 | 0 | 0 |



**Figure S1.** Simulated breeding pattern among adult female free-roaming cat populations. The graph shows the seasonal pattern of reproductive success based on the six-month timestep featured in all simulations.



**Figure S2.** Simulated density dependence in kitten mortality in free-roaming cat populations, where *N* = population size and *K* = local habitat carrying capacity.
